# Target Proteins of Phloretin for Its Anti-Inflammatory and Antibacterial Activities Against *Propionibacterium acnes*-Induced Skin Infection

**DOI:** 10.3390/molecules24071319

**Published:** 2019-04-03

**Authors:** Dasom Cheon, Jieun Kim, Dasom Jeon, Hang-Cheol Shin, Yangmee Kim

**Affiliations:** 1 Department of Bioscience and Biotechnology, Konkuk University, Seoul 05029, Korea; chdasom@konkuk.ac.kr (D.C.); za3524@konkuk.ac.kr (J.K.); dasom921012@konkuk.ac.kr (D.J.); 2 School of Systems Biomedical Science, Soongsil University, Seoul 06978, Korea; hcshin@ssu.ac.kr

**Keywords:** phloretin, plant natural product, *Propionibacterium acnes*, inflammation, antimicrobial activity

## Abstract

Phloretin is a natural chalcone with antibacterial and anti-inflammatory effects. This study investigated the anti-acne activity of phloretin against *Propionibacterium acnes*-induced skin infection and the potential target proteins of its anti-inflammatory and antibacterial effects. Phloretin potently inhibited the growth of *P. acnes* and *P. acnes*-induced Toll-like receptor (TLR) 2-mediated inflammatory signaling in human keratinocytes. Secreted embryonic alkaline phosphatase assay confirmed that the anti-inflammatory activity of phloretin is associated with the *P. acnes*-stimulated TLR2-mediated NF-κB signaling pathway. Phloretin significantly decreased the level of phosphorylated c-Jun N-terminal kinase (JNK), showing a binding affinity of 1.184 × 10^−5^ M^−1^. We also found that phloretin binds with micromolar affinity to *P. acnes* β-ketoacyl acyl carrier protein (ACP) synthase III (KAS III), an enzyme involved in fatty acid synthesis. Conformation-sensitive native polyacrylamide gel electrophoresis showed that phloretin reduced KAS III-mediated 3-ketoacyl ACP production by over 66%. A docking study revealed that phloretin interacts with the active sites of JNK1 and KAS III, suggesting their involvement in *P. acnes*-induced inflammation and their potential as targets for the antibacterial activity of phloretin. These results demonstrate that phloretin may be useful in the prevention or treatment of *P. acnes* infection.

## 1. Introduction

**Figure 1:**
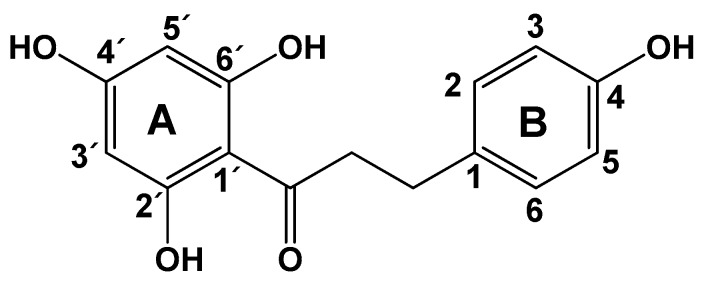
Chemical structure of phloretin (molecular weight: 274.27).

Flavonoids are the most abundant natural products present in foods, such as vegetables, fruits, spices, nuts, red wine, and tea. This large group of secondary plant metabolites includes more than 6000 known compounds and has many biological and pharmacological effects, including anti-inflammatory, antiallergic, antitumor, antibacterial, antiviral, and antioxidant activities [1]. Dietary polyphenols have been widely studied for their potential applications in disease prevention [1]. Phloretin [3-(4-hydroxyphenyl)-1-(2,4,6-trihydroxyphenyl)-1-propanone] is a dihydrochalcone primarily found in apples and strawberries in both free and glucosidic forms (Figure 1) [2,3,4,5], and is known to have antioxidant [6], anticancer [7,8], anti-inflammatory [9], and antibacterial [9,10] activities.

Acne vulgaris is the most common cutaneous disorder of the pilosebaceous unit and is characterized by inflammatory and non-inflammatory lesions that develop mainly on the skin of the face, neck, and chest as a result of infection by the anaerobic gram-positive bacterium *Propionibacterium acnes* (*P. acnes*) [11,12], which was recently renamed *Cutibacterium acnes* (*C. acnes*) based on taxonomic classification [13]. Acne vulgaris lesions include papules, comedones, nodules, and cysts [12]. The immune response to *P. acnes*-induced inflammation plays a critical role in the pathogenesis of acne vulgaris [14]. Many types of medication are administered to treat this condition, including topical agents, oral antibiotics, and oral hormone therapies. Recently, owing to increases in resistance to existing antibiotics and side effects, public interest in natural products for the treatment of acne has increased considerably [15,16]. *P. acnes* contributes to the inflammatory nature of acne by inducing monocytes to produce proinflammatory cytokines, such as tumor necrosis factor (TNF)-α, interleukin (IL)-1β, and IL-8 [17]. *P. acnes* has been reported to activate Toll-like receptor (TLR) 2, which mediates *P. acnes*-induced production of IL-12 and IL-8 by primary human monocytes or IL-6 by macrophages [18,19].

Exposure to *P. acnes* stimulates the production of IL-1α, TNF−α, and granulocyte macrophage colony-stimulating factor by keratinocyte monolayers [20]. The anti-acne effect of phloretin reportedly involves attenuation of cyclooxygenase 2 and prostaglandin E2 expression during *P. acnes*-induced upregulation of inflammatory signaling [21]. In our previous study, the effects of phloretin on human embryonic kidney (HEK)293-hTLR2 cells stimulated with Pam_3_Cys-Ser-(Lys)_4_ (Pam3CSK4) were investigated. The results of the study confirmed that the inhibition of TLR2/1 heterodimerization by phloretin was comparable to that induced by the known TLR2 inhibitor CU-CPT22. Western blotting results showed that phloretin effectively inhibits Pam_3_CSK_4_-induced TLR2 and NF-κB p65 expression and binds to the TLR2–TLR1 interface [22]. However, the detailed mechanism and target proteins involved in the effect of phloretin on *P. acnes*-induced inflammation have not yet been elucidated.

Fatty acids are required for the survival of bacteria; as such, proteins involved in fatty acid synthesis are attractive targets for antibacterial agents. The main difference between fatty acid synthesis in mammals and bacteria is the number of proteins involved. In the former, the process is mediated by a single enzyme, whereas in the latter, there is a different protein at each step [23]; one of these is β-ketoacyl acyl carrier protein (ACP) synthase III (KAS III) [24]. The suppression of fatty acid synthesis in *P. acnes* via inhibition of the activity of KAS III may, therefore, represent an effective antibacterial strategy.

In this study, we investigated the anti-acne effects of phloretin against different strains of *P. acnes*. We also examined the inhibitory effects of phloretin on target proteins involved in *P. acnes*-induced inflammation using secreted embryonic alkaline phosphatase (SEAP) assay, Western blotting, and conformation-sensitive native polyacrylamide gel electrophoresis (PAGE), as well as investigated the interactions between phloretin and target proteins using fluorescence quenching and molecular docking analyses. Our results provide evidence for the potential use of phloretin as a novel and effective anti-acne agent.

## 2. Results

### 2.1. Antimicrobial Activity

We evaluated the antimicrobial activity of phloretin against gram-negative and -positive bacterial strains as compared to that of triclosan and benzoyl peroxide, which were used as positive controls. Triclosan is a well-known antibacterial and antifungal agent used in commercial products, such as shampoo and toothpaste. The U.S. Food and Drug Administration has recently prohibited its use in antibacterial products, such as soaps [25]. Benzoyl peroxide is a topical acne agent that can cause skin reactions, such as dryness, itching, and irritation [26]; as such, many studies have focused on identifying alternatives to this potentially hazardous compound [26,27]. Phloretin showed low antibacterial activity against standard gram-negative and -positive bacterial strains (Table 1). It also showed antibacterial activity against acne-causing drug-resistant *S. aureus*, with an Minimum Inhibitory Concentration (MIC) of 64 μM. Triclosan potently inhibited standard gram-negative and -positive bacterial strains, with MIC values in the range of 0.5 to 1 μM. In contrast, benzoyl peroxide did not exert antibacterial effects on any of the tested strains. We evaluated the activity of phloretin against four different strains of *P. acnes* (Table 1) and found that it had a potent effect against *P. acnes* strains KCTC3220, KCTC5527, and KCTC5933 (MIC = 16 μM) and a weaker effect against strain KCTC3114 (MIC = 32 µM). Triclosan strongly inhibited all four strains of *P. acnes* (MIC = 8 or 16 μM), whereas benzoyl peroxide was far less effective (MIC = 64 μM). These results demonstrate that phloretin has very selective antibacterial activity against *P. acnes* and has stronger antibacterial activity against *P. acnes* than the standard anti-acne agent benzoyl peroxide (Table 1), indicating that it could, therefore, be used as an anti-acne agent.

### 2.2. Cytotoxicity against Mammalian Cells

**Figure 2:**
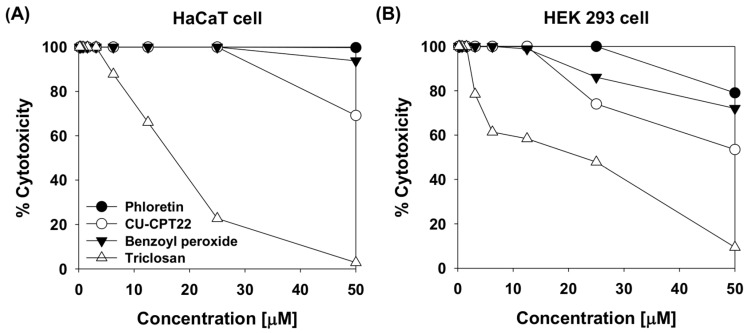
Effects of phloretin, CU-CPT22, benzoyl peroxide, and triclosan on viability of (**A**) HaCaT cells and (**B**) human HEK293 cells.

We investigated the cytotoxicity of phloretin against HaCaT human keratinocytes and human kidney embryonic (HEK)293 cells using Cell Counting Kit-8 (CCK-8). HaCaT cell viability was unaltered by treatment with 50 μM phloretin (Figure 2A). In HEK293 cells, there was a very small change in viability at concentrations over 25 µM, but the dose-response curves showed that the half-maximal inhibitory concentration (IC_50_) values of phloretin were >50 μM (Figure 2A,B) in both cell types. Triclosan was highly toxic to the cells, even at very low concentrations, with an IC_50_ value of 18 μM in HaCaT cells.

A review of the efficacy and safety profiles of triclosan has revealed that it is hazardous to humans, and its toxicity remains under investigation [26,27,28]. CU-CPT22 and benzoyl peroxide were also more toxic than phloretin in HaCaT cells and HEK293 cells. These results suggest that phloretin is safer for use in humans than triclosan, benzoyl peroxide, and CU-CPT22.

### 2.3. Effect of Phloretin on TLR2-Mediated SEAP Activity

**Figure 3:**
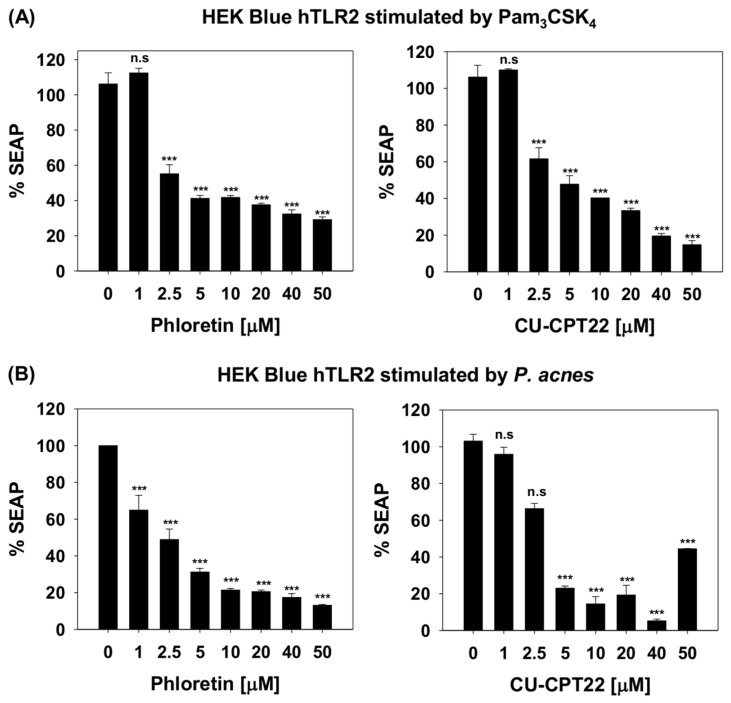
Dose-dependent reduction in secreted embryonic alkaline phosphatase (SEAP) activity reflecting suppression of NF-κB activation in (**A**) 200 ng/mL Pam_3_CSK_4_-stimulated HEK-Blue^®^ hTLR2 cells and (**B**) *P. acnes*-stimulated HEK-Blue^®^ hTLR2 cells. SEAP activities of culture supernatants containing different concentrations of phloretin or CU-CPT22 (0, 1, 2.5, 5, 10, 20, 40, 50 μM) were determined and compared with levels without phloretin or CU-CPT22. All experiments were performed three times independently. *** *p* < 0.001 vs. cells treated with Pam_3_CSK_4_ or *P. acnes* only; n.s, represents no significance.

SEAP assay was used to evaluate the effect of phloretin on TLR2 signaling in HEK-Blue^TM^-hTLR2 cells by monitoring the activation of NF-kB [29]. SEAP assay was performed with phloretin and CU-CPT22 (Hexyl-3,4,6-trihydroxy-2-methoxy-5-oxo-5H-benzo[7]annulene-8-carboxylate) to a maximum concentration of 50 µM before the induction of cytotoxicity. HEK-Blue^TM^-hTLR2 cells were stimulated by a known agonist, Pam_3_CSK_4_, or by *P. acnes*. The results showed that phloretin induced significant concentration-dependent suppression of TLR2-mediated SEAP activity (Figure 3A,B) in cells stimulated with either Pam_3_CSK_4_ or *P. acnes*. When HEK-Blue^TM^-hTLR2 cells were stimulated with Pam_3_CSK_4_, TLR2-mediated reporter activity was enhanced in HEK-Blue^TM^-hTLR2 cells, whereas treatment with phloretin inhibited nearly 50% of the SEAP activity at 2.5 µM. This was comparable to that observed with CU-CPT22. The expression of NF-κB in HEK-Blue^TM^-hTLR2 cells stimulated by *P. acnes* was also suppressed by up to 79% at 10 µM phloretin. These results indicate that phloretin exerts its anti-inflammatory activity via the TLR2-mediated NF-κB signaling pathway in *P. acnes*-induced inflammation.

### 2.4. Measurement of Human (h)IL-1β, hIL-12, and hTNF-α Release by P. acnes-Stimulated HaCaT Cells

**Figure 4:**
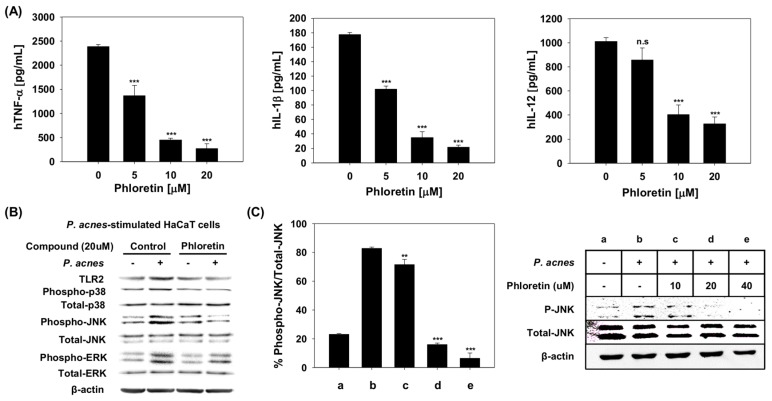
Effects of phloretin on *Propionibacterium acnes*-induced inflammation. (**A**) Effects of phloretin on cytokine levels in *P. acnes*-stimulated HaCaT cells. Tumor necrosis factor (TNF)-α, interleukin (IL)-1β, and IL-12 levels in the culture medium of *P. acnes*-stimulated HaCaT cells were evaluated by enzyme-linked immunosorbent assay. (**B**) Expression of intracellular signaling molecules in *P. acnes*-stimulated HaCaT cells with or without phloretin treatment, as determined by Western blotting; β-actin was used as a loading control. (**C**) Indicated levels of phosphorylation of c-Jun N-terminal kinase (JNK) protein in *P. acnes*-stimulated HaCaT cells with or without phloretin treatment at 0, 10, 20, or 40 μM, quantified using ImageJ. ** *p* < 0.01, *** *p* < 0.001 vs. cells treated with *P. acnes* only. n.s, represents no significance.

Since phloretin inhibits *P. acnes*-induced inflammation via the TLR2-mediated signaling pathway, we next measured the cytokine levels in the culture medium of *P. acnes*-stimulated HaCaT cells (Figure 4A). The results showed that hTNF-α concentrations were 42.6%, 81.1%, and 88.4% lower for cells treated with 5, 10, and 20 μM phloretin, respectively, as compared to those exposed to *P. acnes* without treatment; similar trends were observed for hIL-1β (42.7%, 80.3%, and 87.9% lower, respectively) and hIL-12 (15.3%, 60.1%, and 67.6% lower, respectively).

### 2.5. Effect of Phloretin on Expression of P. acnes-Induced Inflammation-Related Proteins in HaCaT Cells

We investigated the effects of phloretin on *P. acnes*-induced TLR2 expression and the phosphorylation of p38 mitogen-activated protein kinase (MAPK), extracellular signal-related kinase (ERK), and c-Jun N-terminal kinase (JNK) in HaCaT cells by Western blotting. As expected, the expression of TLR2, phospho-p38, phospho-ERK, and phospho-JNK was induced following exposure to *P. acnes*; however, this effect was reduced by phloretin treatment, with TLR2 and phospho-p38, -ERK, and -JNK expression decreasing by 52%, 49%, 36%, and 77%, respectively (Figure 4B). These results showed that pretreatment with phloretin inhibited the *P. acnes*-mediated stimulation of TLR2. Furthermore, it successfully inhibited the phosphorylation of JNK in HaCaT cells. This was confirmed by further assessing the dose-dependent effects of phloretin on the level of phospho-JNK expression. The results showed that the phosphorylation of JNK was inhibited progressively (Figure 3E) as the amount of phloretin increased in HaCaT cells stimulated by *P. acnes;* expression decreased by 13.6%, 80.7%, and 92.2% at phloretin concentrations of 10, 20, and 40 µM, respectively (Figure 4C), compared to the expression level in cells stimulated by *P. acnes* without phloretin.

### 2.6. Binding of Phloretin and JNK1

**Figure 5:**
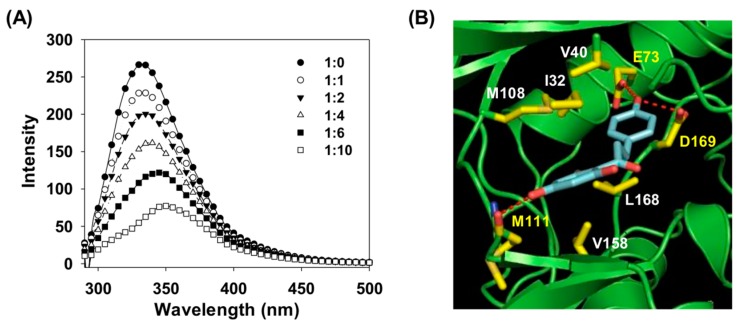
Binding of phloretin to JNK1. (**A**) Fluorescence spectra of JNK1 in the presence of phloretin at the indicated concentrations (pH 7.0). Samples were excited at 290 nm, and emission was recorded at the indicated wavelengths. (**B**) Model of phloretin binding to JNK1. Hydrophobic residues are shown in white, and residues that participate in hydrogen bonding with phloretin are shown in yellow.

To elucidate the mechanism of action of phloretin, we examined the target proteins involved in *P. acnes*-induced inflammation. Since phloretin markedly suppressed phospho-JNK expression in *P. acnes*-stimulated HaCaT cells, we investigated the interaction between phloretin and JNK1, which, along with JNK2, is associated with inflammation, by measuring the binding affinity between these molecules. The dissociation constant for the interaction was determined by fluorescence quenching. The tryptophan fluorescence of JNK1 was reduced in the presence of phloretin (Figure 5A). The binding affinity of phloretin for JNK1 was 1.184 × 10^−5^ M^−1^, indicating a high-affinity interaction.

We next carried out docking studies to simulate the binding of phloretin to the adenosine triphosphate (ATP)-binding pocket (active site) of JNK1. We examined the contribution of hydrogen bonds to the phloretin–JNK1 interaction and found that the 4-hydroxy groups in the phloretin B ring formed an extensive hydrogen bond network with the JNK1 Glu73 and Asp169 side chains, such that the B ring of phloretin was buried in the active site of JNK1. The hydroxy group at the 4′ position of the phloretin A ring formed hydrogen bonds with the backbone amide of Met111. There were additional hydrophobic interactions between the phloretin B ring and Ile32, Val40, and Met111 and between the phloretin A ring and Val158 and Leu168 in JNK1 (Figure 5B).

### 2.7. Activity of P. acnes KAS III

**Figure 6:**
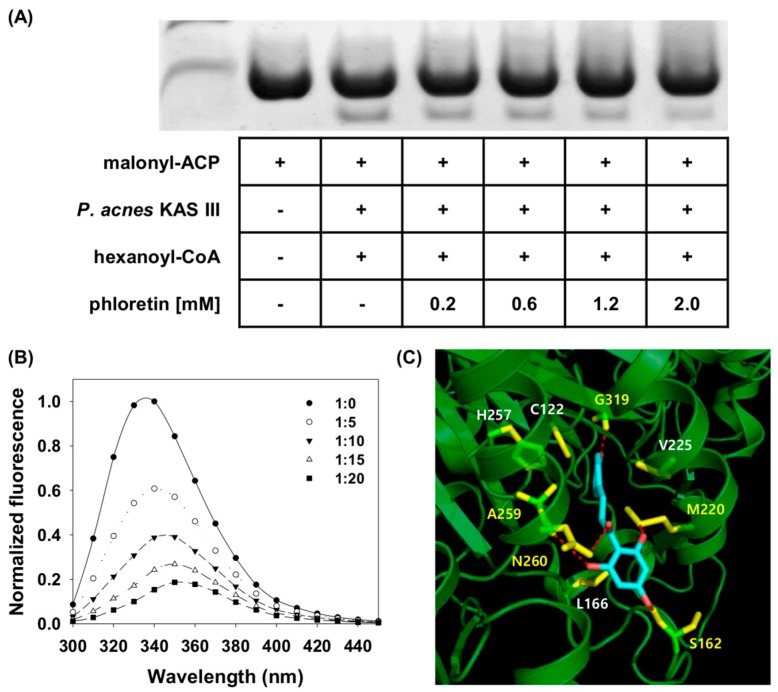
Inhibition of β-ketoacyl acyl carrier protein (ACP) synthase III (KAS III) activity and binding by phloretin. (**A**) Results of conformation-sensitive native polyacrylamide gel electrophoresis (PAGE). Upper and lower bands represent malonyl-ACP and 3-keto hexanoyl-ACP (reaction product), respectively. (**B**) Normalized fluorescence spectra of *P. acnes* KAS III in the presence of indicated concentrations of phloretin. (**C**) Docking pose of phloretin with *P. acnes* KAS III. Hydrophobic residues are shown in white, and residues that participate in hydrogen bonding with phloretin are shown in yellow.

We previously reported the *P. acnes* KAS III structure, which has a distinctive cavity in the active site [30]. Although shorter than that of *Mycobacterium tuberculosis* KAS III, the cavity is large and wide and can accept coenzyme (Co) A with long carbon chains or branched chains. Based on our previous study, we used hexanoyl-CoA as a substrate for conformation-sensitive native PAGE. We performed KAS III inhibition experiments to identify target proteins involved in the antimicrobial activity of phloretin [23]. In the initiation step of elongation, KAS III produces 3-ketoacyl-ACP, CO_2_, and CoA using malonyl-ACP and acyl-CoA (acetyl-CoA in bacteria). Malonyl-ACP and hexanoyl-CoA were used as substrates to induce the KAS III reaction in vitro. The product, 3-keto-hexanoyl-ACP, showed shifts on the gel (Figure 6A). These results indicate that phloretin suppresses the activity of *P. acnes* KAS III.

### 2.8. Interaction of Phloretin and P. acnes KAS III

Fluorescence quenching experiments were performed to evaluate the binding of phloretin to *P. acnes* KAS III (Figure 6B). In our previous study, we measured the binding affinity between acyl-CoA and KAS III as 10^−4^ M^−1^ [30]. Here, we found that the binding affinity of phloretin to *P. acnes* KAS III was 10^−7^ M^−1^, implying that phloretin can inhibit the activity of *P. acnes* KAS III by binding to its active site with a much higher affinity than the substrate.

A docking study was carried out to confirm the binding between *P. acnes* KAS III and phloretin (Figure 6C). The results showed that hydrogen bonding occurred between the Asn260 of *P. acnes* KAS III and the ketone group next to the A ring of phloretin. Ser162, Met220, and Ala259 of KAS III also had hydrogen bonds with the hydroxyl groups of the A ring of phloretin, while Gly319 exhibited hydrogen bonding with the hydroxyl group of the phloretin B ring. Hydrophobic interactions were observed between Leu166 of KAS III and the A ring of phloretin and between Cys122, Met220, Val225, His257, and Ala259 of KAS III and the B ring of phloretin. Cys122, Val225, Ala259, His257, and Asn260 are conserved in the KAS III proteins of most bacteria. The cysteine and histidine residues are particularly important, as they belong to the catalytic triad [31] and are involved in acyl group transfer from acyl-CoA and the reaction between the acyl group and malonyl-ACP. In the docking model, phloretin interacted with this cysteine residue of *P. acnes* KAS III, suggesting this as the mechanism of acyl-CoA inhibition. Thus, phloretin binds to the active site of *P. acnes* KAS III and blocks the elongation reaction between *P. acnes* KAS III and its substrates.

## 3. Discussion

Polyphenols are secondary plant metabolites involved in defense against environmental stress and pathogenic microbes and have various biological effects as well as health benefits [32]. Phloretin, a dihydrochalcone flavonoid, is a natural product abundant in apples and strawberries and is a common constituent of daily diet in humans [33]. In this study, we demonstrated that phloretin can directly or indirectly alleviate pathogenic *P. acnes* infection by suppressing the bacterial growth and activation of associated inflammatory signaling pathways. Phloretin also has very selective potent antibacterial activity against *P. acnes.* The activity of phloretin against *P. acnes* strains was more potent (16–32 μM) than that of the widely used anti-acne agent benzoyl peroxide. Triclosan, which served as a positive control in this study, is now banned in soap due to its cytotoxicity, although it was formerly a common antibacterial ingredient in many cleaning and cosmetic products [25]. A recent study in mice showed that triclosan weakened the contractility of cardiac and skeletal muscles [34]. We found here that triclosan and benzoyl peroxide were both toxic to human HaCaT cells and HEK293 cells, whereas 50 μM phloretin did not significantly affect cell viability (Figure 2A,B). Thus, phloretin may be a natural and effective substitute for triclosan in consumer products.

*P. acnes* induces the secretion of proinflammatory cytokines, such as TNF-α, IL-1α, IL-1β, IL-6, and IL-8 by mononuclear cells [35,36]. IL-1β is abundantly expressed in inflammatory acne lesions [37], and increased production of IL-12 has been detected following exposure to heat-killed *P. acnes* [38]. In acne lesions, TLR2-expressing macrophages surround pilosebaceous follicles, and *P. acnes* induces cytokine production by monocytes via TLR2 [19]. *P. acnes* also initiates an inflammatory response involving the production of various cytokines, including IL-1α, IL-1β, IL-8, and TNF-α, by keratinocytes via TLR2 [20,39,40]. *P. acnes*-induced inflammatory responses are attenuated by anti-TLR2-neutralizing antibodies and fully blocked by CU-CPT22 [41]. Since bacterial infection by *P. acnes* usually accompanies inflammatory acne lesions, it is important to find anti-acne agents that have antibacterial as well as anti-inflammatory activities. Phloretin was previously reported to have an anti-inflammatory effect in lipopolysaccharide-stimulated RAW264.7 cells [10], which involved blockade of nuclear translocation of the NF-κB subunit p65 and of MAPK phosphorylation [10,33].

Our previous study showed that phloretin inhibits the heterodimerization of TLR2/1 and that the binding affinity of phloretin to TLR2 was 8.3 × 10^-6^ M, underscoring the possibility of targeting Pam_3_CSK_4_-induced TLR2 signaling in the treatment of TLR2-mediated inflammatory immune diseases [22]. In this study, SEAP activity was significantly inhibited by phloretin in *P. acnes*-stimulated HEK-Blue^®^hTLR2 cells, indicating that phloretin inhibited NF-κB activation in *P. acnes*-stimulated HEK-Blue^®^hTLR2 cells. Furthermore, exposure of *P. acnes*-stimulated HaCaT cells to phloretin significantly reduced JNK activation. These findings demonstrate that phloretin suppresses *P. acnes*-induced inflammation via TLR2-mediated inflammatory signaling. Phloretin decreased the phosphorylation of JNK in *P. acnes*-stimulated HaCaT cells in a dose-dependent manner; we also demonstrated that the hydroxy group at position 4 of the phloretin B ring plays a key role in its interaction with JNK1, which is critical for the inhibitory effect of phloretin.

KAS III was found to be a possible target protein for the antibacterial activity of phloretin. In our previous study, we reported that phloretin has anti-tuberculosis activity against *M. tuberculosis* KAS III [42], which has high sequence homology to *P. acnes* KAS III [30]. The results of conformation-sensitive native PAGE showed that phloretin inhibits the reaction of *P. acnes* KAS III with its substrates, decreasing the amount of product. In addition, fluorescence quenching experiments showed that phloretin has high affinity (2.769 × 10^−7^ M^−1^) for *P. acnes* KAS III. In docking studies, the phloretin and *P. acnes* KAS III models showed that the active site residues of KAS III interact with the A and B rings of phloretin; more specifically, the latter interacts with the catalytic Cys122 of KAS III, which blocks the reaction between *P. acnes* KAS III and its substrates.

In conclusion, our study demonstrates that phloretin suppresses *P. acnes*-induced inflammation by inhibiting JNK via TLR2-mediated inflammatory signaling. Phloretin binds with high affinity to and blocks the activity of *P. acnes* KAS III, resulting in the inhibition of fatty acid synthesis, which is required for the survival of *P. acnes*. Thus, phloretin is a less toxic and more potent alternative to triclosan and benzoyl peroxide for the treatment of *P. acnes*-induced skin infection.

## 4. Materials and Methods

### 4.1. Phloretin and Chemicals

Phloretin was purchased from Sigma-Aldrich (St. Louis, MO, USA); the purity was determined to be 99% by high-performance liquid chromatography (HPLC) and mass spectrometry (Korea Basic Science Institute, Ochang, Korea). Phloretin was dissolved in dimethyl sulfoxide to obtain a 10 mg/mL stock solution. Triclosan with a purity of 97% was purchased from Bio Basic Inc. (Markham, Ontario, Canada). Benzoyl peroxide was determined to have a purity of 99.9% using HPLC (Korea Basic Science Institute, Ochang, Korea).

### 4.2. Antibacterial Activity of Phloretin

We purchased *Escherichia coli* (KCTC1682), *Salmonella typhimurium* (KCTC 1926), *Staphylococcus aureus* (KCTC 1621), *Bacillus subtilis* (KCTC 1021), and *Propionibacterium acnes* (KCTC 3314, KCTC 3220, KCTC 5527, and KCTC 5933) from the Korean Collection for Type Cultures (KCTC), Korea Research Institute of Bioscience & Biotechnology (Daejeon, Korea). *S. aureus* (CCARM 0027 and CCARM 3708) was obtained from the Culture Collection of Antimicrobial Resistant Microbes (Seoul, Korea). The MIC of phloretin against each of these bacteria was determined by broth microdilution assay [43]. We defined the MIC as the lowest concentration of phloretin that completely inhibited bacterial growth; this was calculated as the average of three independent measurements in the range of 0.5 to 512 µM. *P. acnes* was grown for 48 to 72 h at 37 °C on sheep blood agar under anaerobic conditions in a mixture of H_2_ and CO_2_ (95:5, *v/v*). Microbroth dilution using Muller–Hinton (MH) broth was used to determine the efficacy of phloretin against *P. acnes.* We diluted a bacterial suspension in MH broth to achieve turbidity equivalent to a McFarland standard of 0.5 (~1 × 10^8^ cells/mL), yielding 1 × 10^4^ cells/mL in each well after inoculation. Phloretin MICs were determined after incubation for 48 h at 37 °C.

### 4.3. Culture of HaCaT, HEK293, and HEK-Blue^®^ hTLR2 Cells

HaCaT cells were cultured using Dulbecco’s modified Eagle’s medium (DMEM) with 1% penicillin-streptomycin and 10% fetal bovine serum. HEK293 cells were cultured in the same way, except 100 µg/mL normocin was added. In the case of HEK-Blue^®^ hTLR2 cells (hkb-htlr2; InvivoGen, San Diego, CA, USA), 1× HEK selection and 100 µg/mL normocin were added to the medium. All cells were incubated at 37 °C in a 5% CO_2_ humidified incubator.

### 4.4. Cytotoxicity of Phloretin Against Mammalian Cells

The cytotoxicity of phloretin was evaluated in HaCaT and HEK293 cells using CCK-8 (Abbkine Scientific, Wuhan, China). Cell viability was determined by measuring absorbance at 450 nm with a microplate reader and is expressed as the average of triplicate measurements from three independent experiments [44].

### 4.5. Enzyme-Linked Immunosorbent Assay

*P. acnes* (KCTC3314) was grown on sheep blood agar for 72 h at 37 °C under anaerobic conditions (H_2_ and CO_2_; 95:5, *v*/*v*). HaCaT cells were treated with phloretin, triclosan, and benzoyl peroxide for 1 h before stimulation with *P. acnes* for 16 h at 37 °C in a 5% CO_2_ incubator. Antibodies against TNF-α, IL-12, and IL-1β were immobilized on immune plates and used for enzyme-linked immunosorbent assay (ELISA; R&D Systems, Minneapolis, MN, USA) [45]. Results are presented as mean ± standard deviation of at least three independent experiments.

### 4.6. Effect of Phloretin on TLR2-Mediated SEAP Activity

HEK-Blue^®^ hTLR2 cells (InvivoGen) were seeded in 96-well plates (density of 5 × 10^4^ cells/well) with phloretin in HEK-Blue detection medium (InvivoGen). After 1 h, cells were treated with Pam_3_CSK_4_ (200 ng/mL) or *P. acnes* (1 × 10^4^ cells/well). SEAP activity was determined after 16 h by measuring absorbance at 630 nm with an ELISA reader.

### 4.7. Western Blotting

HaCaT cells were pretreated with phloretin for 1 h before stimulation by *P. acnes* (1×10^8^ cells/mL) for 20 min. Proteins were isolated from *P. acnes*-stimulated HaCaT cells cultured in the presence or absence of phloretin, and Western blotting was performed as previously described [45,46]. We quantified the relative signal intensity of protein bands using ImageJ software v1.8.0 (National Institutes of Health, Bethesda, MD, USA).

### 4.8. Measurement of Binding Affinity by Fluorescence Quenching

The C-terminal truncated form of human JNK1α1 (residues 1–364) was cloned into the pET21b expression vector (Novagen, Billerica, MA, USA) and expressed in *E. coli* with a 6His tag at the C-terminus. JNK1 was purified as previously reported [47]. *P. acnes* KAS III was cloned into the pET15b expression vector (Novagen), and Cys122 of *P. acnes* KAS III was mutated to Ala122 to enhance the solubility of the protein. *P. acnes* KAS III C122A was expressed, purified and used to measure the binding constant of phloretin, which was compared to that of acyl-CoA and *P. acnes* KAS III C122A [30]. Phloretin was titrated against 10 μM JNK1 or 3 μM *P. acnes* KAS III solution, and fluorescence quantum yields were calculated by measuring tryptophan emission on a RF-5301PC Spectrofluorophotometer (Shimadzu, Kyoto, Japan) [48].

### 4.9. Conformation-Sensitive Shift Assay

Native PAGE was performed using a 22% Tricine gel to confirm the activity of wild-type *P. acnes* KAS III. Malonyl-ACP (1.8 nmol), hexanoyl-CoA (0.3 nmol), and *P. acnes* KAS III (0.06 nmol) were mixed with phloretin (2, 6, 12, or 20 nmol) in a total volume of 10 μL. The reaction was performed at room temperature for 25 min [30].

### 4.10. Docking Studies

The X-ray crystallographic structure of free-form JNK1 (pdb entry: 3V3V.pdb) was used for phloretin binding studies [35]. Phloretin was docked in JNK1 using the CDOCKER protocol in Discovery Studio v4.1 (BIOVIA, San Diego, CA, USA) [49]. The docking model of *P. acnes* KAS III (6A9N.pdb) complexed with phloretin was obtained using the same protocol. Molecular dynamics-simulated annealing was carried out using a rigid protein and flexible ligand; the final minimization step was applied to the docking pose of the ligand [49,50].

### 4.11. Statistical Analyses

At least three independent samples were included in each analysis. Data were analyzed using InStat v3.05 software (GraphPad, La Jolla, CA, USA). Values were considered statistically significant at *p* < 0.05.

## Figures and Tables

**Table 1:** Antimicrobial activities of phloretin.

Bacterial Strain	Minimum Inhibitory Concentration (μM)		
	Phloretin	Triclosan	Benzoyl Peroxide
**Standard Bacteria**			
*Escherichia coli* (KCTC1682)	512	<0.5	>512
*Salmonella typhimurium* (KCTC1926)	512	<0.5	>512
*Staphylococcus aureus* (KCTC1621)	128	<0.5	>512
*Bacillus subtilis* (KCTC1021)	128	1	>512
**Acne-Causing Strains**			
*S. aureus* (CCARM0027)	64	0.5	64
*S. aureus* (CCARM3708)	64	0.5	64
***Propionibacterium acne* Strains**			
*P. acnes* (KCTC3314)	32	8	64
*P. acnes* (KCTC3220)	16	8	64
*P. acnes* (KCTC5527)	16	16	64
*P. acnes* (KCTC5933)	16	16	64
